# Data relating to threats to passion fruit production in the Neotropics due to agricultural area loss and pollinator mismatch as consequence of climate changes

**DOI:** 10.1016/j.dib.2019.103802

**Published:** 2019-03-05

**Authors:** Antonio Diego M. Bezerra, Alípio J.S. Pacheco Filho, Isac G.A. Bomfim, Guy Smagghe, Breno M. Freitas

**Affiliations:** a Universidade Federal Do Ceará, Departamento de Zootecnia, Setor de Abelhas, Bloco 814 – Av. Mister Hull, 2977, Fortaleza, Ceará, Brazil; b Ghent University, Department of Plants and Crops, Coupure Links 653, 9000, Ghent, Belgium

## Abstract

The data in this article are associated with the research article ‘Agricultural area losses and pollinator mismatch due to climate changes endanger passion fruit production in the Neotropics’ (A.D. Bezerra et al. 2019).

The data consists of the occurrence points, AUC scores models, presence and absence and co-occurrence maps of the passion fruit (*Passiflora edulis*) crop and its pollinators, *Xylocopa* bees (*Xylocopa frontalis* and *Xylocopa grisescens*), in current and future scenarios (RPC 4.5 and 8.5, in the years 2060 and 2080) in the Neotropics. Data was obtained though literature review (articles, systematic surveys, dissertation and thesis), as well as systematic searches in entomological collections available in data portals provided by the *SpeciesLink* and *Global Biodiversity Information Facility* – GIBF, and analyses by the MaxEnt algorithm and binary transformation. Occurrence error points that did not represent the actual spatial distribution of the species were removed to obtain the current occurrence points and data analyses proved good performance of models for all prediction scenarios. The data-generated maps of pollinators and crop occurrence and co-occurrence also show how climate change may impact the spatial distribution of pollinators and potential losses of this crop's agricultural areas.

**Table:** Specifications table

Subject area	Agriculture
More specific subject area	Climate change
Type of data	Table, figure
How data was acquired	Literature review (articles, systematic surveys, dissertation and thesis), as well as systematic searches in entomological collections available in data portals provided by the *SpeciesLink* and *Global Biodiversity Information Facility* – GIBF.
Data format	Filtered and Analyzed
Experimental factors	Cleaning and extraction of the occurrence errors points that did not represent the actual spatial distribution of these species
Experimental features	Data analyses of spatial distribution of the crop and bees using the MaxEnt algorithm
Data source location	The Neotropics
Data accessibility	All data are presented in the paper
Related research article	A.D.M. Bezerra, A.J.S. Pacheco Filho, I.G.A. Bomfim, G. Smagghe, B.M. Freitas Agricultural area loss and pollinator mismatch due to climate changes endanger passion fruit production in the Neotropics, Agricultural Systems, In Press [1].

**Table:** 

**Value of the data** • The data present AUC score models and maps of occurrence and co-occurrence for pollinators and the passion fruit crop in the Neotropics for the current and future scenarios of climate changes in 2060 and 2080. • The data can be used by researchers and policy makers to assist studies and policies regarding the implications of crop-pollinator mismatch and losses of agricultural areas; • These are the first data showing the current and future effects of climate change acting concomitantly on a tropical crop and its key pollinators. • The data show losses of adequate area for the crop and the pollinators over time and increasing crop-pollinator mismatch.

## 1 Data

**Fig. 1:**
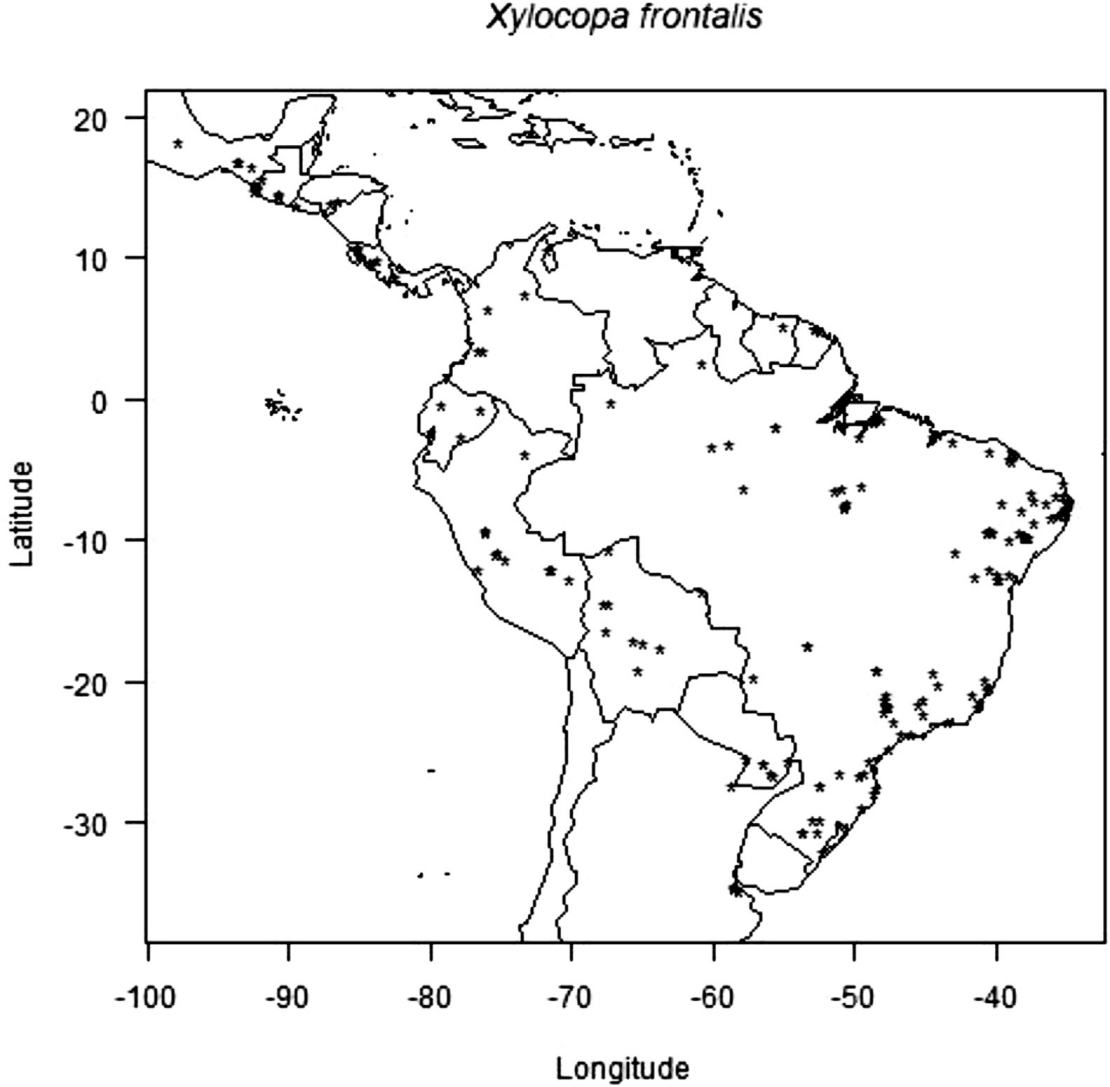
Occurrence points of *Xylocopa frontalis* in the Neotropics.

**Fig. 2:**
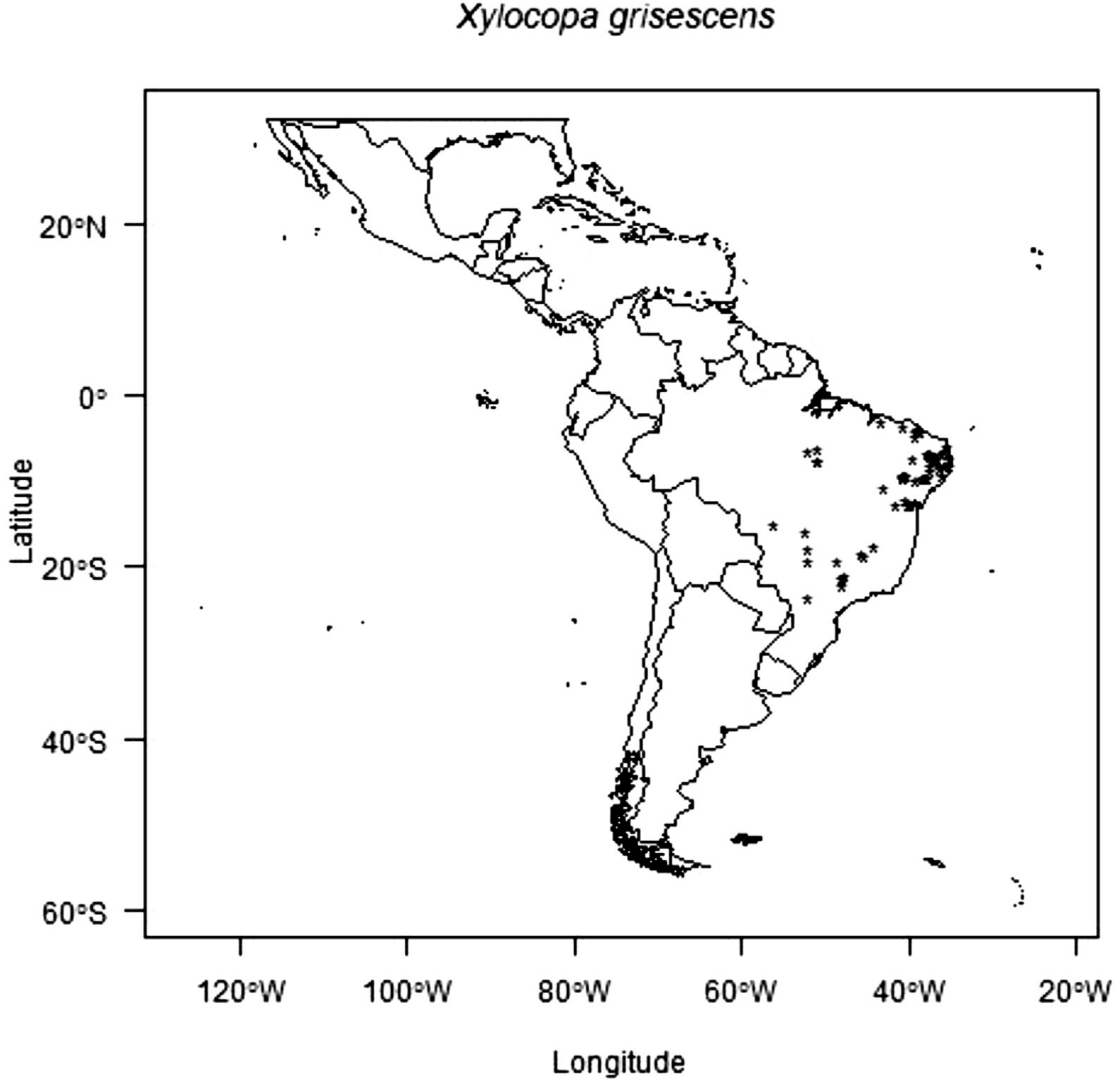
Occurrence points of *Xylocopa grisescens* in the Neotropics.

**Fig. 3:**
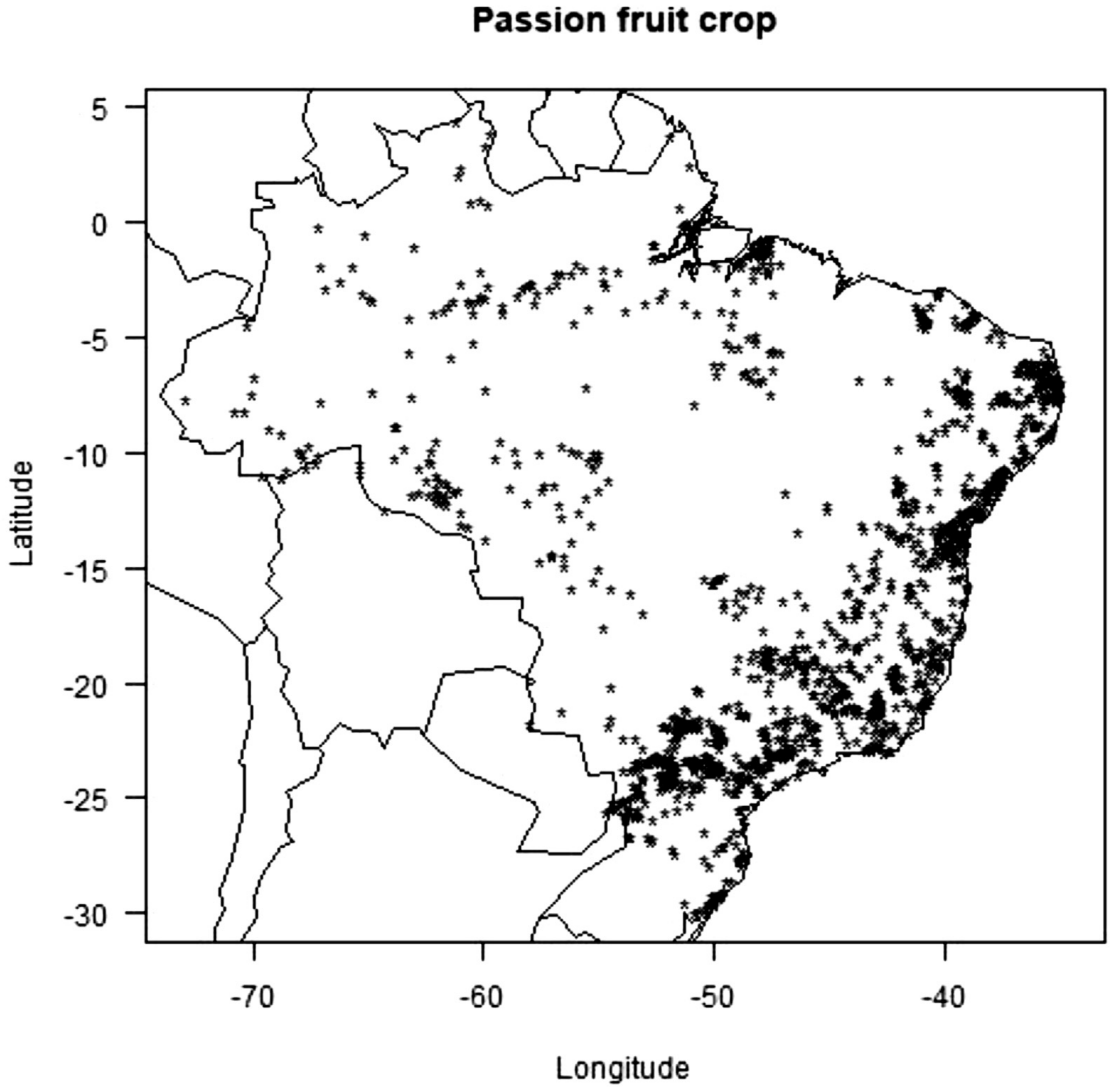
Centroids of 1195 Brazilian counties growing passion fruit.

**Fig. 4:**
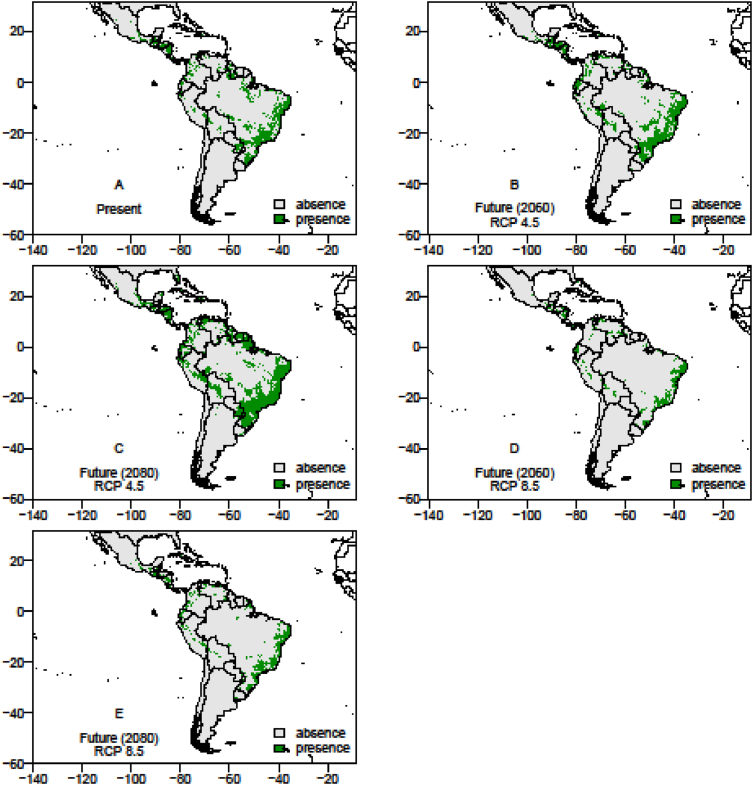
The presence and absence maps in the current, moderate (RCP 4.5) and pessimistic (RCP 8.5) scenarios in years 2060 and 2080, showing the spatial distribution area of *Xylocopa frontalis* in the Neotropics. In the futures pessimistic scenarios the range of species presence area reduced 47.95% (RCP 8.5, 2060) and 29.03% (RCP 8.5, 2080). In moderate scenarios the area which this species will change range show an increment up to 59.70%. The potential lost area for *X. frontalis* is estimated between 15.48% and 57.71% in the future scenarios.

**Fig. 5:**
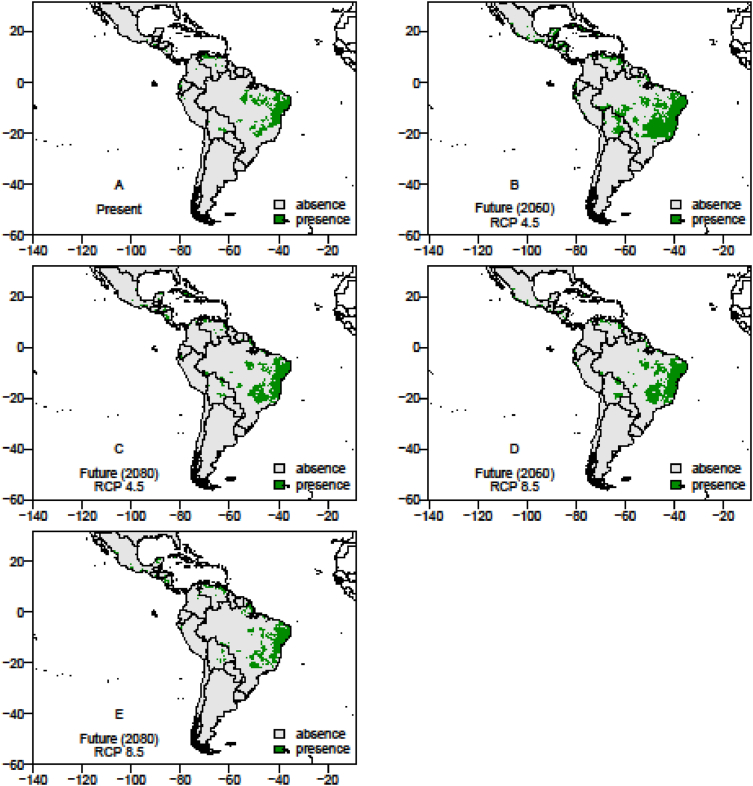
The presence and absence maps in the current, moderate (RCP 4.5) and pessimistic (RCP 8.5) scenarios in years 2060 and 2080 show the spatial distribution area of *Xylocopa grisescens* in the Neotropics. In the futures scenarios, the potential lost area for this species is estimated in 15.41% (RCP 4.5, 2060) and 27.81% (RCP 4.5, 2080), in moderate scenarios and 23.52% (RCP 8.5, 2060) and 35.32% (RCP 8.5, 2080) in pessimistic scenarios. In moderate scenarios the area which this species will change range show an increment up to 115.02%.

**Fig. 6:**
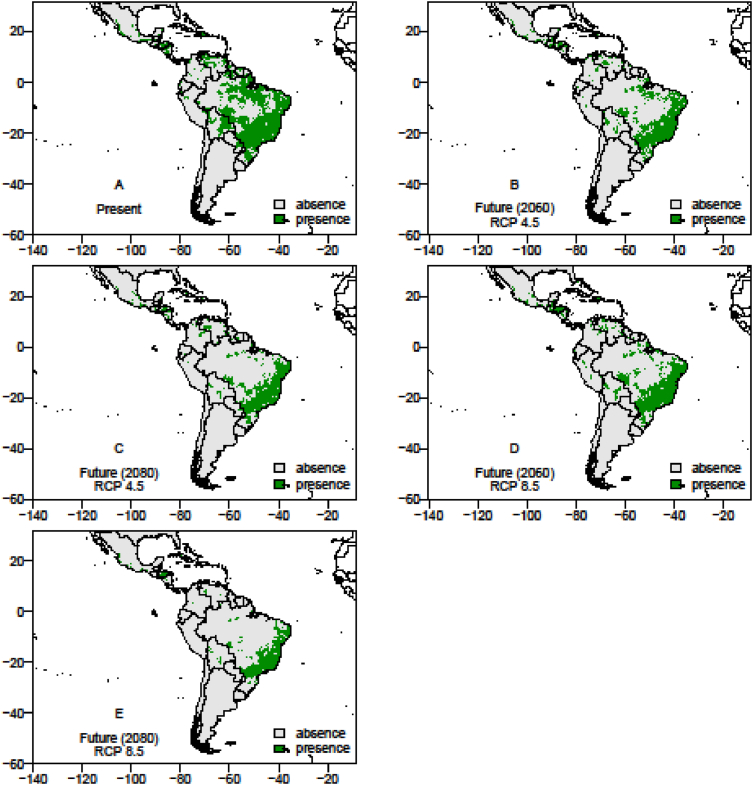
The presence and absence maps in the current, moderate (RCP 4.5) and pessimistic (RCP 8.5) scenarios in years 2060 and 2080 show the suitable area for growing passion fruit (*Passiflora edulis* Sims f. *flavicarpa*) in the Neotropics. All models show that climate change will be jeopardizing passion fruit crop due to the future climatic conditions, which will reduce the suitable cropping areas of passion fruit orchards. When comparing the current scenarios with the futures scenarios for 2060 and 2080 the suitable areas for the passion fruit crop will be reduced in all scenarios, mainly in the pessimistic one (RCP 8.5; 2080) with reductions up to 63.67%%. The potential area losses for cropping passion fruit may be between 42.90% and 64.86%.

**Fig. 7:**
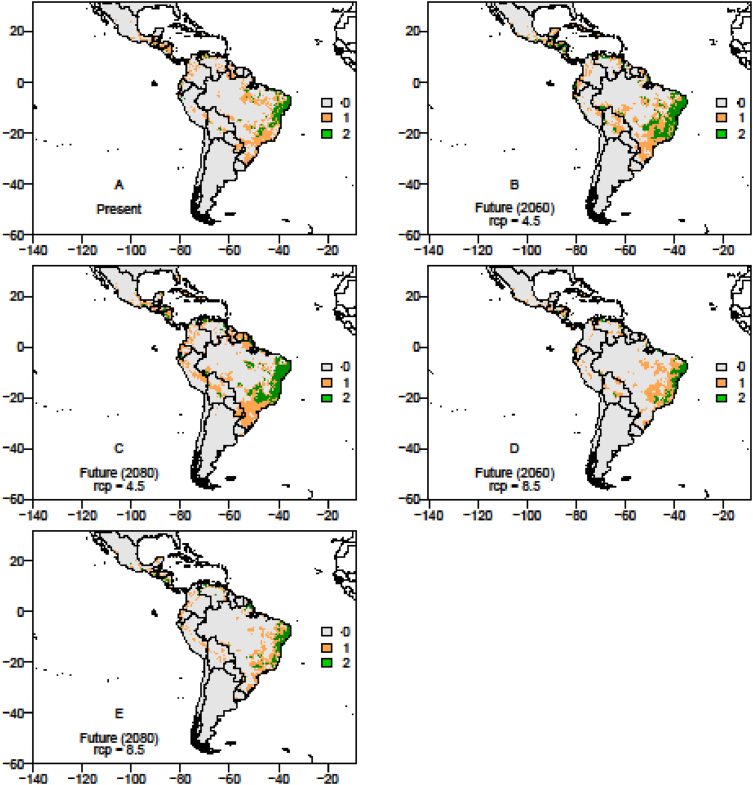
The overlapping map of *Xylocopa* bees in the Neotropics, showing the spatial distribution range in the current, moderate (RCP 4.5) and pessimistic (RCP 8.5) scenarios in years 2060 and 2080. *X*. *frontalis* potential occurrence area: Current scenario = 761,755.5 km^2^; RCP 4.5 (2060) scenario = 1,216,566.0 km^2^, RCP 4.5 (2080) scenario = 396,499.5 km^2^; RCP 8.5 (2060) scenario = 396,499.5 km^2^; RCP 8.5 (2080) scenario = 540,616.5 km^2^; *X. grisescens* potential occurrence area: Current scenario = 410,148.0 km^2^; RCP 4.5 (2060) scenario = 881,883.0 km^2^; RCP 4.5 (2080) scenario = 580,590.0 km^2^; RCP 8.5 (2060) scenario = 597,145.5 km^2^; RCP 8.5 (2080) scenario = 493,533.0 km^2^. Legends: 0 – no species occurrence area; 1 – one species occurrence area; 2- two species occurrence area.

**Fig. 8:**
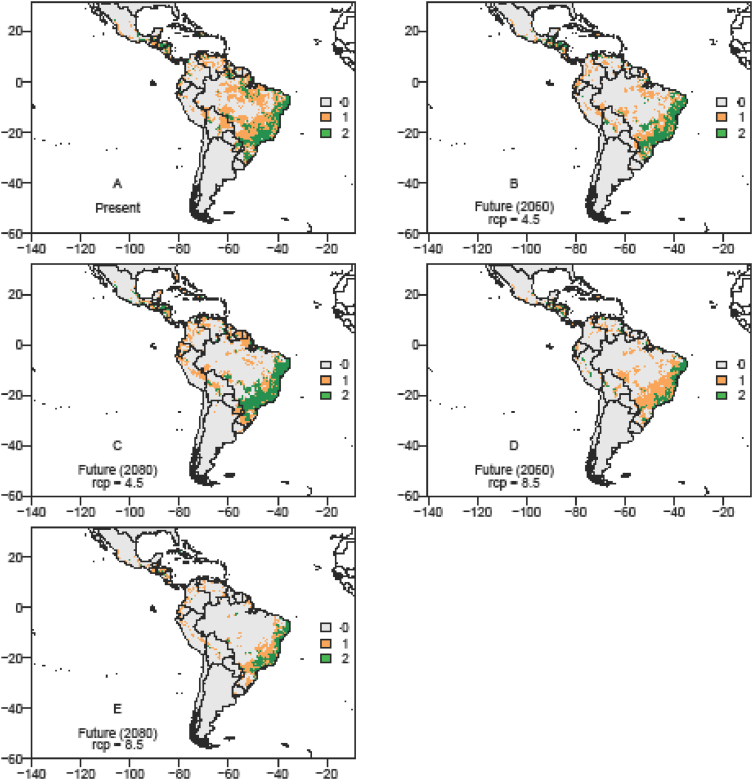
The overlapping map of *Xylocopa frontalis* and passion fruit crop in the Neotropics, showing the suitable area range in the current, moderate (RCP 4.5) and pessimistic (RCP 8.5) scenarios in year 2060 and 2080. The suitable area for passion fruit crop potential occurrence: Current scenario = 1,469,151.0 km^2^; RCP 4.5 (2060) scenario = 895,765.5 km^2^; RCP 4.5 (2080) scenario = 765,355.5 km^2^; RCP 8.5 (2060) scenario = 932,044.5 km^2^; RCP 8.5 (2080) scenario = 533,700.0 km^2^; *X*. *frontalis* potential occurrence area: Current scenario = 761,755.5 km^2^; RCP 4.5 (2060) scenario = 1,216,566.0 km^2^, RCP 4.5 (2080) scenario = 396,499.5 km^2^; RCP 8.5 (2060) scenario = 396,499.5 km^2^; RCP 8.5 (2080) scenario = 540,616.5 km^2^. Legends: 0 – no species occurrence area; 1 – one species occurrence area; 2- two species occurrence area.

**Fig. 9:**
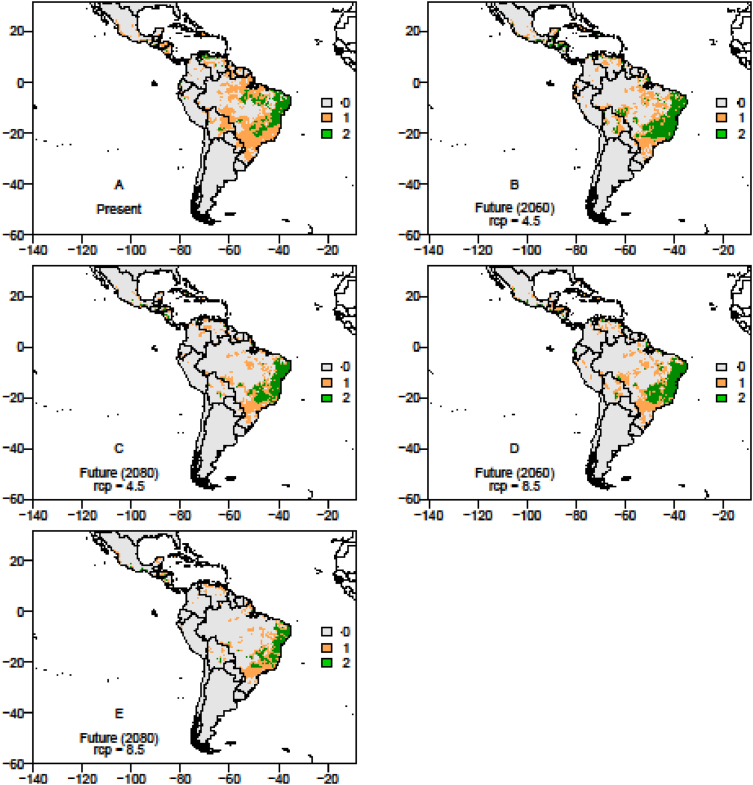
The overlapping map of *Xylocopa grisescens* and passion fruit crop in the Neotropics, showing the suitable area range in the current, moderate (RCP 4.5) and pessimistic (RCP 8.5) scenarios in year 2060 and 2080. The suitable area for passion fruit crop potential occurrence: Current scenario = 1,469,151.0 km^2^; RCP 4.5 (2060) scenario = 895,765.5 km^2^; RCP 4.5 (2080) scenario = 765,355.5 km^2^; RCP 8.5 (2060) scenario = 932,044.5 km^2^; RCP 8.5 (2080) scenario = 533,700.0 km^2^; *X. grisescens* potential occurrence area: Current scenario = 410,148.0 km^2^; RCP 4.5 (2060) scenario = 881,883.0 km^2^; RCP 4.5 (2080) scenario = 580,590.0 km^2^; RCP 8.5 (2060) scenario = 597,145.5 km^2^; RCP 8.5 (2080) scenario = 493,533.0 km^2^. Legends: 0 – no species occurrence area; 1 – one species occurrence area; 2- two species occurrence area.

The data on records of occurrence points for the passion fruit pollinators, *Xylocopa* bees (*X. frontalis* and *X. grisescens*), were obtained through literature review, systematic surveys, articles, dissertations and theses, as well as any information available in data portals provided by the *SpeciesLink* and *Global Biodiversity Information Facility* – GIBF and the centroids of 1195 Brazilian counties where passion fruit has been grown for the past ten years (see spreadsheet in supplementary material). After removing duplicated data, incomplete information or incorrect coordinates, these data showed the actual occurrence points of *Xylocopa* bees and the passion fruit crop in the Neotropics (Fig. 1, Fig. 2 and Fig. 3). To the spatial distribution modelling, 19 layers of each scenario from *WorldClim* was used in our analyses, each layer having a spatial resolution of 2.5 arc minutes (Cells with size ∼4.5 km resolution at the equator). The spatial distribution modelling analyses of all three species produced AUC scores close to 1.0 indicating good performances of the models’ prediction (Table 1). More information about the importance of bioclimatic variables is available in Ref. [1]. The maps of species occurrence and co-occurrence indicate relevant effects of climate change in species shift, potential area losses and crop-pollinator mismatch in the future scenarios (Figs. 4–9) (see Fig. 6) (see Fig. 7) (see Fig. 8) (see Fig. 5).

**Table 1:** The scores of the Area Under the receiver operating characteristic Curve (AUC) and cutoff (threshold) to Maximum Entropy (MaxEnt) for each species *Xylocopa frontalis* and *Xylocopa grisescens* and passion fruit (*Passiflora edulis* Sims f. *flavicarpa*).

Scenarios		Species		
		*Xylocopa frontalis*	*Xylocopa grisescens*	*Passiflora edulis*
Current	AUC	0.9245	0.9475	0.9218
	Cutoff	0.4235	0.2579	0.1957
RCP 4.5 (2060)	AUC	0.9111	0.9614	0.8981
	Cutoff	0.3864	0.1433	0.2834
RCP 4.5 (2080)	AUC	0.9222	0.9425	0.9172
	Cutoff	0.2865	0.2016	0.2995
RCP 8.5 (2060)	AUC	0.9222	0.9621	0.9024
	Cutoff	0.5355	0.1756	0.2566
RCP 8.5 (2080)	AUC	0.9122	0.9689	0.9124
	Cutoff	0.4824	0.2189	0.4043

## 2 Experimental design, materials and methods

### 2.1 Biotic and climate data

Data on sampling points of the most common pollinators of passion fruit flowers in Central and South Americas, the carpenter bee species *X. frontalis* Oliver, 1789 and *X. grisescens* Lepeletier, 1841 were analyzed for the occurrence and absence of these bee species in the Neotropical region [2], [3], [4].

Records of occurrence points for both bee species were obtained through literature reviews, as well as systematic searches in entomological collections available in data portals such as the *Specieslink* and *Global Biodiversity Information Facility* – GIBF. The data generated 371 occurrence points to *X. frontalis* and 188 occurrence points to *X. grisescens* (see spreadsheet in supplementary material). Occurrence points found in duplicated, incomplete information or incorrect coordinates were removed [5], and the remaining data showed the actual occurrence points of *Xylocopa* bees in the Neotropics. Passion fruit coordinate points were obtained in the County Agricultural Production database available from the Brazilian Institute of Geography and Statistics (IBGE) website. The centroids of 1195 counties where passion fruit was cultivated in the last ten years were used as the basis to point coordinates [6].

Layers of bioclimatic variable for current and futures scenarios were obtained through *Worldclim* [7], each layer having a spatial resolution of 2.5 arc minutes (Cells with size ∼4.5 km resolution at the equator). A total of 19 layers were used in all scenarios. We obtained forecasts for the future climatic conditions considering the scenarios RCP 4.5 and RCP 8.5 (Representative Concentration Pathways), in 2060 and 2080 developed by Hadley Center Global Environmental Model (HadGEM2-ES)) [8]. Pearson's correlation coefficient and Principal Components Analysis (PCA) were used for each pairwise comparison of the 19 climatic variables to estimate the correlation levels between bioclimatic variables. The most explanatory variable was selected according to PCA, only when the bioclimatic variable presented strong correlation (r≥|0.75|) with two or more variables. The number of bioclimatic variables left in the models is available in Ref. [1].

### 2.2 Model building and current and future occurrence

The Species Distribution Modelling (SDM) was developed using the package Dismo v.1.1–4 [9], R language [10]. For this, we used the ecological niche modelling applying the MaxEnt (Maximum entropy) algorithm [11]. The high AUC scores indicated that the model is based on information, if compared to a random model without information, which the AUC score was 0.50. The AUC scores (close to 1.0) indicated a good performance of the models (Table 1).

A random set of pseudo-absence data to each species was required to make the current and futures scenarios maps to the whole study area (Latitude, 32° East to −60° West; Longitude, 24° North to −125° South). We determined the threshold (cutoff) with Dismo v1.1-4 package [9], and with Biomod2 v.3.3–7 package [12] in R language [10], and we perform a binary transformation for each models, the current and future scenario of each map was compared. We also compared the passion fruit map scenarios with *X. frontalis* and *X. grisescens*. It was possible to show the suitable areas for each species in the current scenario and the changes in the spatial distribution range of the species in the future scenarios, as well as the overlap area suitable for crop and for the bee species.
